# Endovascular management of proximal ovarian artery perforation near the ostium during uterine fibroid embolization

**DOI:** 10.1016/j.jvscit.2026.102213

**Published:** 2026-03-02

**Authors:** Yi-Wei Wu, Enming Yong, Terrence Hui, Gabriel Chan

**Affiliations:** a Department of Diagnostic Radiology, Tan Tock Seng Hospital, Singapore; b Department of General Surgery, Tan Tock Seng Hospital, Singapore

**Keywords:** Ovarian, Fibroid, Uterine artery embolization, Stents

## Abstract

Ovarian artery injury during uterine fibroid embolization is rare but can result in significant hemorrhage, especially when the injury occurs near the arterial ostium. We describe a case of iatrogenic proximal ovarian artery perforation near the ostium complicated by retroperitoneal hematoma in a patient with severe thrombocytopenia owing to underlying aplastic anemia. Definitive hemorrhage control was achieved using distal coil embolization of the injured ovarian artery combined with covered aortic stent graft deployment across the ostium. This case highlights the technical considerations, risk factors, and an effective endovascular salvage strategy for managing ovarian arterial injuries near the aorta.

Iatrogenic arterial injury is an uncommon but potentially serious complication of endovascular procedures. Ovarian artery perforation during uterine fibroid embolization is rare, with periostial perforation being even less common and more challenging to manage. We report a case of proximal ovarian artery perforation near the ostium complicated by retroperitoneal hemorrhage, successfully managed with distal coil embolization and aortic covered stent graft deployment. The patient is deceased. Consent for publication was obtained from patient's next of kin.

## Case report

A 58-year-old woman with a background of aplastic anemia presented with severe vaginal bleeding in the setting of a large uterine fibroid. On admission, she had profound pancytopenia (hemoglobin, 5 g/dL; platelet count, 33 × 10^9^/L). Surgical management was deemed high risk, and she was referred for uterine fibroid embolization.

**Fig 1:**
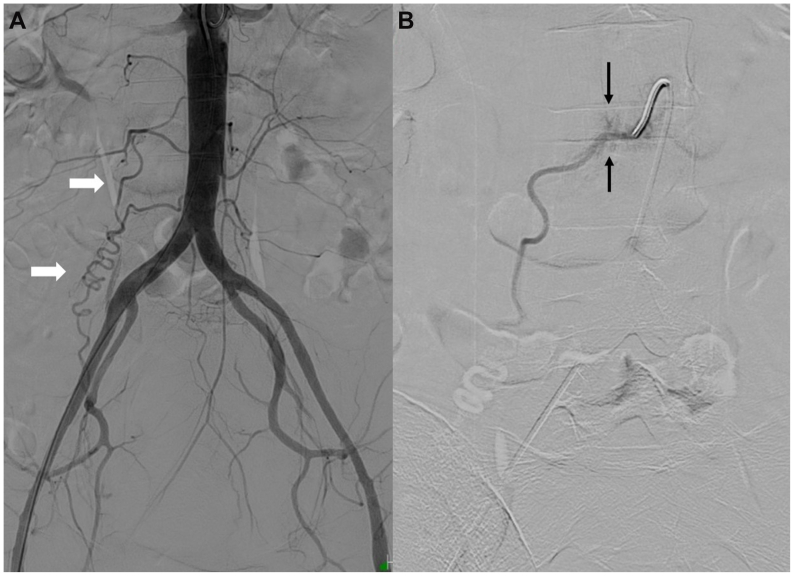
**(A)** Flush aortogram demonstrated tortuous right ovarian artery (*white arrows*) that supplied the uterine fibroid. **(B)** Selective ovarian artery angiogram showed evidence of rupture near the ostium (*black arrows*).

The procedure was performed under local anesthesia and moderate sedation via right common femoral arterial access using a 5F sheath. Bilateral uterine arteries were successfully embolized. Subsequent aortography demonstrated additional supply to the uterine fibroid from the tortuous right ovarian artery (Fig 1, *A*). The ostium of the right ovarian artery was engaged using a 5F Shepherd Hook catheter (Cordis). Multiple attempts at selective cannulation with a 2.7F microcatheter and microwire (Transend, Boston Scientific) were unsuccessful. A repeat angiogram demonstrated contrast extravasation near the origin of the right ovarian artery (Fig 1, *B*), raising concern for arterial perforation. The patient developed acute back pain and tachycardia, although blood pressure remained stable.

**Fig 2:**
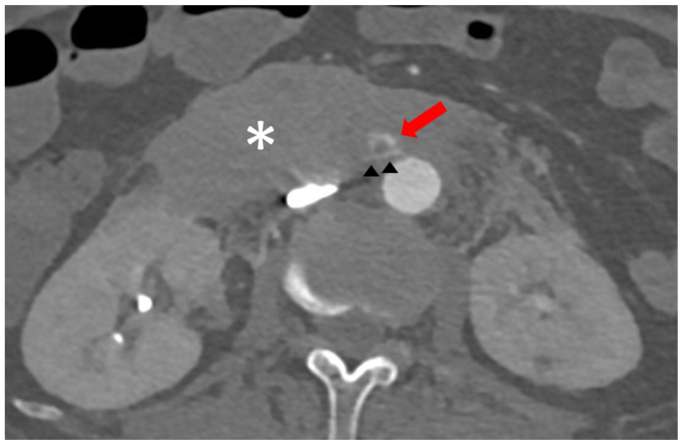
Computed tomography scan showing contrast extravasation near the ostium of the ovarian artery (*red arrow*) with large retroperitoneal hematoma (∗). There was sharp angulation of the right ovarian artery arising from the aorta (*black arrowheads*).

**Fig 3:**
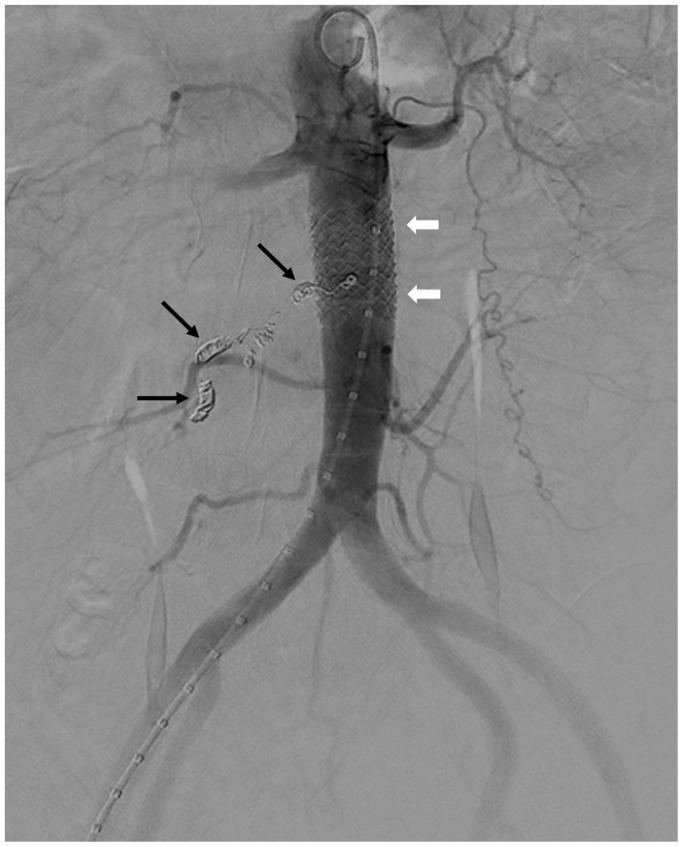
Final aortogram showed coils in the ovarian artery (*black arrows*) and aortic stent graft (*white arrows*) with no evidence of further bleeding.

Urgent computed tomography scan confirmed rupture of the right ovarian artery near its ostium (Fig 2), with a large retroperitoneal hematoma. Note was made of sharp angulation of the ovarian artery arising from the aorta (Fig 2). Given the patient's thrombocytopenia and ongoing hemorrhage, emergent endovascular intervention was deemed necessary. The right femoral access was upsized to an 11F sheath after preclosure. The right ovarian artery was selectively catheterized using a softer 0.014-inch guidewire (PT^2^, Boston Scientific) with low tip load in combination with a smaller 2.0F microcatheter (Truselect, Boston Scientific). The 5F Shepherd Hook catheter was used to gently hook the ostium without deep engagement, allowing improved alignment and more working space for the microcatheter wire system to enter the vessel. Coil embolization was performed to prevent retrograde bleeding (Fig 3). To definitively exclude the arterial origin and prevent ongoing bleeding, a 16 × 38 mm aortic covered stent graft (BeGraft, Bentley InnoMed) was deployed across the ovarian artery origin (Fig 3).

Hemodynamic stability was restored, and vaginal bleeding improved after the procedure. Hemoglobin levels subsequently recovered to 10 g/dL, and a follow-up computed tomography scan performed 1 week later demonstrated near-complete resolution of the retroperitoneal hematoma. Subsequently, hemoglobin levels and platelet counts again declined, attributed to progression of the patient's underlying aplastic anemia, necessitating more frequent blood transfusions. The patient later developed neutropenic sepsis, with suspected infection involving the infarcted uterine fibroid. The patient was transferred to a national specialist center for further management of the uterine mass and aplastic anemia, including consideration of hysterectomy, approximately 5 weeks after the procedure. Owing to institutional/government data protection regulations, detailed clinical information after transfer were not accessible. Based on available information, the patient died 9 weeks after the index procedure at another institution. Given the interval of 9 weeks between the procedure and death, together with interval imaging demonstrating resolution of the retroperitoneal hematoma, the cause of death was considered unlikely to be related to the salvage endovascular procedure.

## Discussion

Iatrogenic injury to the ovarian artery during uterine fibroid embolization is rare but can result in significant retroperitoneal hemorrhage, particularly when the injury occurs near the arterial ostium. In the present case, perforation near the ovarian artery origin led to active bleeding in a patient with known hematological disease with severe thrombocytopenia. Definitive hemorrhage control was achieved using a combined endovascular strategy of distal coil embolization and covered stent graft deployment across the aortic ostium.

Although the uterine arteries are the primary targets during uterine fibroid embolization, embolization of ovarian arterial supply may be required in select cases to achieve adequate control of fibroid-related bleeding. Ovarian artery contribution is more frequently encountered in patients with large fundal fibroids or prior pelvic or gynecological surgery.^1^ Failure to recognize or treat ovarian artery supply may result in persistent symptoms or incomplete treatment,^2^ underscoring the importance of identifying ovarian arterial feeders when clinically indicated.

Wire-related arterial perforations are often self-limited; however, severe complications may occur depending on the anatomical location of the injury and patient-specific factors.^3^ Injuries near the arterial ostium are of particular concern because of proximity to the aorta and the higher risk of uncontrolled hemorrhage. This risk is further amplified in patients with thrombocytopenia or coagulopathy, in whom even limited vascular injury may have significant clinical consequences.

Selective cannulation of the ovarian artery should, therefore, be performed with caution. Repeated or forceful wire manipulation should be avoided when selective cannulation is difficult. The use of low tip-load guidewires commonly employed in peripheral vascular interventions (eg, a PT^2^ wire) may reduce the risk of intimal injury during catheterization.^4^ In addition, preprocedural cross-sectional imaging can provide important anatomical information. The identification of sharp or acute ostial angulation should prompt consideration of modified catheter configurations, including creating a cleft by cutting a small portion of the catheter tip to facilitate catheterization.^5^ In situations such as the present case, where the ovarian artery originates from the aorta with an acute right-sided turn, removal of a small portion of the right side of the catheter tip creates a directional cleft. This allows the microwire and microcatheter to exit laterally through the cleft rather than the end hole, facilitating smoother entry into the acutely angulated branch. By improving coaxial alignment, this technique may reduce the risk of intimal injury or perforation near the ostium.

In the literature, ovarian artery rupture has most commonly been reported in association with aneurysmal disease during pregnancy or related to large fibroids.^6^ These cases are typically managed with transarterial embolization alone, using coils to achieve proximal and distal (front door and back door) occlusion of the aneurysmal segment. Isolated reports have also described ovarian artery injury related to surgical or obstetric procedures, which were similarly treated by transarterial embolization.^7^ In contrast, iatrogenic guidewire perforation of the ovarian artery near the aortic ostium represents a distinct clinical scenario, in which embolization alone may be insufficient because of the inadequate coverage of the inflow directly from aorta. To our best knowledge, severe retroperitoneal hemorrhage resulting from guidewire perforation of the ovarian artery during uterine fibroid embolization has not been described previously.

In such situations, distal coil embolization of the injured branch combined with covered stent graft deployment across the ostium provides effective exclusion of the injured segment and definitive hemorrhage control. This approach allows preservation of aortic integrity while avoiding open surgical repair, particularly in high-risk patients.

## Conclusions

Combined distal coil embolization and aortic covered stent grafting is an effective endovascular salvage strategy for ovarian artery ostial injury complicated by hemorrhage.

## Funding

None.

## Disclosures

None.
